# Highly Water Dispersible Functionalized Graphene by Thermal Thiol-Ene Click Chemistry

**DOI:** 10.3390/ma14112830

**Published:** 2021-05-25

**Authors:** Farzaneh Farivar, Pei Lay Yap, Tran Thanh Tung, Dusan Losic

**Affiliations:** 1 School of Chemical Engineering and Advanced Materials, The University of Adelaide, Adelaide, SA 5005, Australia; farzaneh.farivar@adelaide.edu.au (F.F.); peilay.yap@adelaide.edu.au (P.L.Y.); tran.tung@adelaide.edu.au (T.T.T.); 2 ARC Hub for Graphene Enabled Industry Transformation, The University of Adelaide, Adelaide, SA 5005, Australia

**Keywords:** graphene, functionalized graphene, thiol-ene click reaction, dispersible graphene

## Abstract

Functionalization of pristine graphene to achieve high water dispersibility remains as a key obstacle owing to the high hydrophobicity and absence of reactive functional groups on the graphene surface. Herein, a green and simple modification approach to prepare highly dispersible functionalized graphene via thermal thiol-ene click reaction was successfully demonstrated on pristine graphene. Specific chemical functionalities (–COO, –NH_2_ and –S) on the thiol precursor (L-cysteine ethyl ester) were clicked directly on the sp^2^ carbon of graphene framework with grafting density of 1 unit L-cysteine per 113 carbon atoms on graphene. This functionalized graphene was confirmed with high atomic content of S (4.79 at % S) as well as the presence of C–S–C and N–H species on the L-cysteine functionalized graphene (FG-CYS). Raman spectroscopy evidently corroborated the modification of graphene to FG-CYS with an increased intensity ratio of D and G band, I_D_/I_G_ ratio (0.3 to 0.7), full-width at half-maximum of G band, FWHM [G] (20.3 to 35.5) and FWHM [2D] (64.8 to 90.1). The use of ethanol as the reaction solvent instead of common organic solvents minimizes the chemical hazards exposure to humans and the environment. This direct attachment of multifunctional groups on the surface of pristine graphene is highly demanded for graphene ink formulations, coatings, adsorbents, sensors and supercapacitor applications.

## 1. Introduction

Graphene materials have sparked enormous attraction and interest from both the scientific and industrial community owing to the fascinating properties of graphene and its widespread applications across many technological fields, such as nanoelectronics, sensors, composites, coatings, batteries, supercapacitors and hydrogen storage [1]. The ongoing demand for graphene and its products is reflected in the rapid wave of graphene technologies transferring from the laboratory to the marketplace [2]. Despite the outstanding optical, electrical, and thermal conductivity properties of graphene, it is no secret that there are still limitations associated with the poor water dispersibility and low intrinsic reactivity of graphene, which hamper its useful applications [2,3,4,5]. To address these limitations, functionalization of pristine graphene with organic functional groups to obtain stable dispersions in various solvents is a vital move to produce graphene materials with new properties [4]. Different strategies including covalent and non-covalent approaches were explored and successfully used for graphene functionalization [4].

Covalent attachment of organic functional groups to the graphene surface can be achieved either by chemical reaction of free radicals or dienophiles sp^2^ carbons of pristine graphene, or by the formation of a covalent bond between organic molecules and the oxygen groups of graphene oxide, GO [4]. The radical-mediated thiol-ene reaction that directly attacks the sp^2^ carbon in the graphene framework appears to be a promising covalent functionalization approach that offers several benefits. It is highly efficient, simple to execute, insensitive to oxygen and water, has no side products and proceeds rapidly with high yield [6]. Several examples in the literature have showcased the application of thiol-ene click functionalization on GO, including our recent studies that demonstrated the use of thiol-ene modified GO with cysteamine and pentaerythritol tetrakis(3-mercaptopropionate) through both thermal and photoinitiated approaches for the removal of water pollutants [7,8,9,10,11,12]. Thiol-ene click reaction has been proven for the chemical modification of graphene oxide (GO), and the thiol-ene clicked GO serves as an excellent host matrix for platinum nanoparticles for catalytic and sensing applications [12]. In another example, one-step thiol-ene click reaction by mercaptosuccinic acid was used to prepare graphene quantum dots (GQDs) decorated with a number of carboxyl groups on the surface which improved their dispersibility in water [13].

Surprisingly, up to now, the majority of the reports of functionalized graphene have primarily focused on GO and its reduced form, reduced graphene oxide (rGO), where the preparation methodologies usually involve the use of hazardous chemicals and toxic gases, with large quantities of chemical waste generated after the reactions [4]. To perform direct covalent functionalization on pristine graphene, having an inert surface and fewer defects, poses considerable challenges and requires high energetic species to break its sp^2^ honeycomb framework. In fact, limited reports were found on the direct functionalization of pristine graphene via thiol-ene click chemistry. Castelain et al. used thiol-ene reaction for a direct functionalization of the graphene surface with short chain polyethylene (PE) brushes which were successfully used to prepare graphene based high-density polyethylene nanocomposites [14]. Peng et al. also used microwave-assisted thiol-ene click reaction for functionalization of graphene using thiol precursors with different functional groups [15]. In another study, cysteamine hydrochloride was bonded to the graphene surface via thiol-ene click chemistry to immobilize Au nanoparticles as an efficient electrochemical sensor [16]. Although these studies have shown that thiol-ene click functionalization can be successfully performed on pristine graphene, there is a research gap regarding the safe use of solvents, including highly volatile organic and non-environmentally friendly solvents such as N-methylpyrrolidone, N,N-dimethylformamide or ortho-dichlorobenzene. Ensuring dispersibility of graphene in these solvents during modification reactions remains a challenging issue. Moreover, the efficiency of thiol-ene click functionalization on pristine graphene is still not satisfactorily accomplished compared to the thiol-ene modification achieved by GO and rGO.

**Figure 1:**
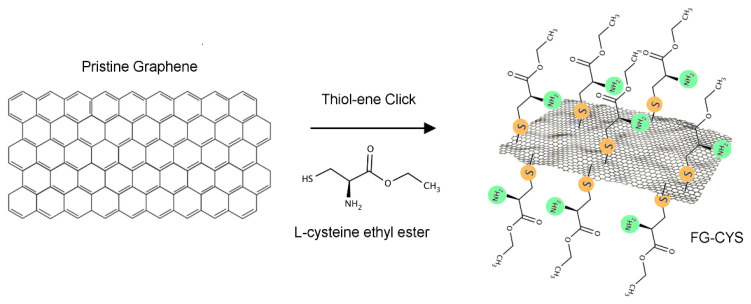
Schematic diagram of the green functionalization of pristine graphene using L-cysteine ethyl ester via thiol-ene click reaction.

Herein, we present a simple, green and sustainable method to functionalize pristine graphene through thiol-ene click reaction to acquire water dispersible graphene materials with multiple functional groups that are important for many applications. The aims of this work are twofold: first, to improve an existing thiol-ene click reaction using more sustainable and green conditions for the functionalization of pristine graphene, and second, to demonstrate versatility of this method to generate graphene with multifunctional surface chemistry with different end groups including ester, amino, and thioether. The developed method is based on the covalent attachment of thiol molecules on the sp^2^ carbon of graphene via thermal thiol-ene click reaction, which is schematically presented in Figure 1. The method is highly efficient, catalyst-free, simple, with mild reaction conditions for surface modifications, which may provide scalable production of the functional graphene materials. To eliminate the hazardous conditions commonly found in thiol-ene click reaction based on toxic solvents, herein, we introduce for the first time the use of ethanol/water mixture (70/30), which is less expensive, non-toxic and more environmentally friendly, to improve the scalability of highly dispersible functionalized graphene materials. A broad range of characterization techniques such as Raman spectroscopy, Fourier-transform infrared spectroscopy (FTIR), X-ray photoelectron spectroscopy (XPS), thermogravimetric analysis (TGA), and water dispersibility test were used to confirm the thiol-ene click functionalization.

## 2. Materials and Methods

### 2.1. Materials and Chemicals

Graphene was obtained from a local company (First Graphene pty ltd, Perth, WA, Australia). L-cysteine (Sigma-Aldrich, Sydney, NSW, Australia), thionyl chloride (Sigma-Aldrich, Sydney, NSW, Australia), diethyl ether (RCI Labscan, Bangkok, Thailand) 2,2′-azobis-(2-methylpropionitrile) (AIBN, Sigma-Aldrich, Sydney, NSW, Australia,), ethanol (Chem-Supply, Adelaide, SA, Australia) were used directly without prior purification. High purity milli-Q water (18.2 MΩ·cm^−1^) was used throughout the work, unless otherwise stated.

### 2.2. Synthesis of L-Cysteine Ethyl Ester

Ethyl esters of cysteine were synthesized according to a reported method [17]. In brief, L-cysteine was first suspended in ethanol, and cooled to 5 °C. Thionyl chloride was added gradually, over a period of 20 min, and the reaction mixture was stirred at room temperature for 5 h. Dry ether was then added to the solution until an opaque solution appeared. The mixture was kept in the refrigerator for a few hours for crystallization of ethyl ester hydrochloride. The final product was collected by filtration. 

### 2.3. Synthesis of Functionalized Graphene (FG-CYS)

Functionalized graphene with L-cysteine ethyl ester (FG-CYS), was prepared according to the adapted procedure reported by Yap et al. [8]. In a typical procedure, 50 mg graphene powder was first dispersed in 100 ml ethanol–water mixture 70% (*v*/*v*) via sonication for 1 h. Subsequently, the sonicated mixture was purged with nitrogen gas for 30 min to create an inert environment. Then, L-cysteine ethyl ester and 2,2′-azobis-(2-methylpropionitrile) (AIBN) were added into the mixture with a further nitrogen gas purge for 30 min, followed by a 30 min sonication. After that, the reaction mixture was poured into a round bottom flask with an additional 30 min of nitrogen gas purge and heated in an oil bath at 65 °C under reflux conditions overnight. After the reaction, the as-synthesized product was washed thoroughly with ethanol and deionized water using centrifuge, dried in oven at 65 °C overnight, and stored for further characterization.

### 2.4. Characterizations

FTIR spectra were collected at 500 to 4000 cm^−1^ on a Nicolet 6700 Fourier Transform Infrared (FTIR) Spectrometer (Thermo Fisher Sci, Sydney NSW, Australia). Morphology of the materials was imaged using a scanning electron microscope (FE-SEM, Quanta 450 FEG, FEI, USA) at an operating voltage of 10 kV and a transmission electron microscope at 120 kV (TEM, FEI Tecnai G2 Spirit, FEI, USA; Philips CM200, Japan at 200 kV). Chemical composition and chemical species were analyzed by X-ray Photoelectron Spectroscopy (XPS, AXIS Ultra DLD, Kratos, UK) equipped with a monochromatic Al Kα radiation source (hv = 1486.7 eV) at 225 W, 15 kV and 15 mA. XPS survey scans were performed at 0.5 eV step size over −10 to 1100 eV at 160 eV pass energy with peak fitting analysis executed using Casa XPSTM software. The core-level XPS spectra were calibrated at 284.8 eV. Raman spectrometer (LabRAM HR Evolution, Horiba Jvon Yvon Technology, Kyoto, Japan) with an excitation wavelength of 532 nm (mpc 3000 laser source) was applied from 500 to 3000 cm^−1^ with an integration time of 10 s for three accumulations to determine the vibrational features of pristine graphene, and thermogravimetric analysis (TGA) of functionalized graphene was performed at a heating rate of 10 °C/min in nitrogen atmosphere from 25 to 1000 °C on a METTLER TOLEDO TGA/DSC 2 instrument.

## 3. Results

**Figure 2:**
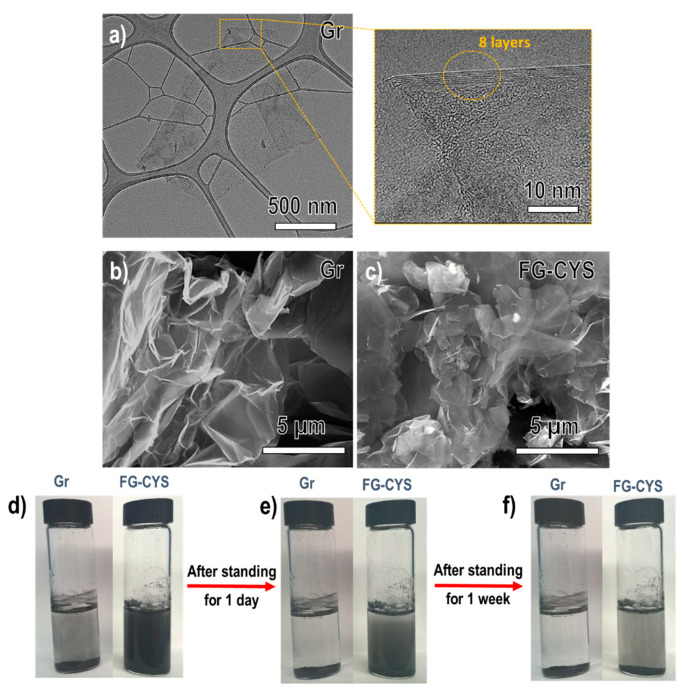
(**a**) TEM image of pristine graphene (Gr) with an enlarged image of HRTEM analysis showing 8 layers of graphene sheets, FESEM of (**b**) Gr and (**c**) FG-CYS. Dispersion test in water (0.5 mg/mL) from left to right: pristine graphene, functionalized graphene with cysteine ethyl ester (FG-CYS) for standing after (**d**) 2 h, (**e**) 1 day and (**f**) 1 week of free-standing.

The morphology of pristine graphene and the functionalized graphene was first examined using scanning (SEM) and transmission (TEM) electron microscopy, as shown in Figure 2a–c. Large and folded few-layer graphene (FLG) sheets, as depicted in Figure 2a, were observed under TEM analysis with an inset showing fewer than 10 layers of graphene used as the precursor in this work. A detailed statistical distribution analysis (Figure S1) indicates an average of six layers of graphene, confirming the FLG used in this study. Under the SEM, pristine graphene (Figure 2b) exhibited highly crumpled wrinkled thin sheets. After the modification using the thiol precursor, the primitive thin and crumpled graphene sheets still remained in FG-CYS, with its surface decorated with multiple fluffy clusters, as shown in Figure 2c. The presence of these fluffy clusters on the surface of graphene sheets preliminarily suggested the effective attachment of L-cysteine ethyl ester moieties on the surface of graphene. 

A simple and rapid water dispersion test was performed to examine the effectiveness of thiol-ene functionalization (Figure 2d–f). As expected, pristine graphene showed poor dispersion, with its powder sedimented immediately after two hours of ultrasonication. The complete sedimentation of the graphene powder at the bottom of the bottle (Figure 2d–f) could be attributed to its low surface activity and weak bonding strength with the water matrix. In contrast, the thiol-ene functionalized graphene FG-CYS) visibly showed good dispersion in water after modification with thiol precursor, as depicted in Figure 2d–f. Remarkably, FG-CYS showed a stable aqueous dispersion in water even after standing for one week (Figure 2f). The enhanced dispersion of the functionalized graphene in water compared to pristine graphene suggested an effective attachment of polar functional groups, such as oxygen groups including ester and amino groups, from the thiol precursor to the graphene surface that rendered the interaction with the polar solvent (water) primarily through hydrogen bonds.

**Figure 3:**
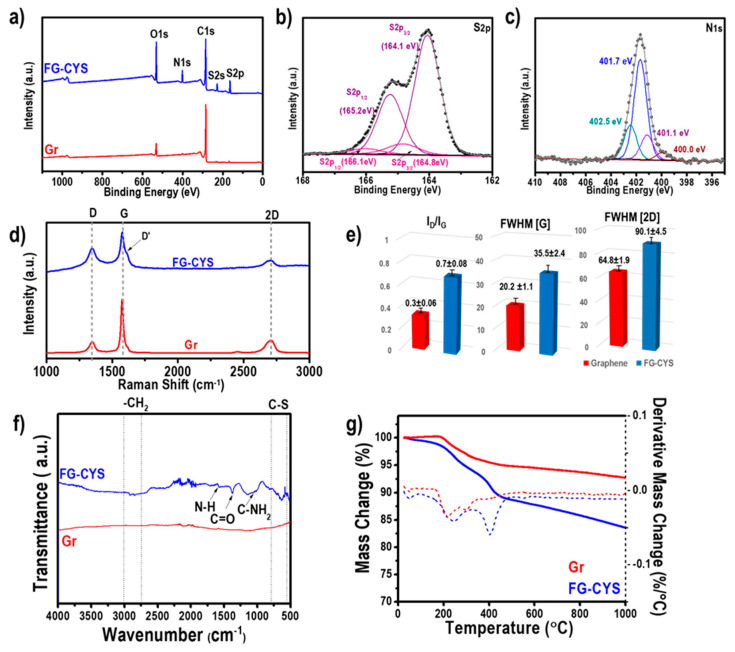
(**a**) XPS survey spectra of Gr and FG-CYS, deconvoluted high-resolution XPS spectra of (**b**) S2p and (**c**) N1s of Gr and FG-CYS, (**d**) Raman spectra, (**e**) I_D_/I_G_, FWHM of Raman G and FWHM of Raman 2D peak for Gr and FG-CYS, (**f**) FTIR spectra and (**g**) TGA-DTG thermograms in N_2_ atmosphere of Gr and FG-CYS.

Chemical composition of the functionalized graphene was determined by XPS analysis (Table 1) with their survey spectra incorporated in Figure 3a. Significant appearance of S2p and N1s peaks, as well as the elevated intensity of O1s peaks of FG-CYS at binding energies of 164.0 eV, 401.5 eV and 531.0 eV, respectively, were observed in the survey scan of FG-CYS relative to pristine graphene. A notably increased atomic concentration of nitrogen (6.26 at %) and sulfur (4.79 at %) on FG-CYS, relative to pristine graphene, implied successful grafting of thiol precursor on the surface of graphene. As depicted in Figure 3b, the doublet peaks of S2p_3/2_ and S2p_1/2_ at around 164.1 eV and 165.2 eV, respectively, can be assigned to thioether sulfur bonded to carbon (C–S–C) species on the narrow XPS spectra of S2p of FG-CYS, and confirmed the successful attachment of the thiol groups from the thiol precursor via thiol-ene click reaction on the surface of the graphene [18,19]. Meanwhile, S2p_3/2_ and S2p_1/2_ peaks with lower intensities deconvoluted at 164.8 eV and 166.1 eV, respectively, could be ascribed to C–S or S–S species on the surface of graphene [20]. Note that S2p peak, associated with highly oxidized S species (>166 eV), was absent from the high-resolution S2p spectrum of FG-CYS, which implied good stability of the sulfur species formed on the surface of graphene sheets, despite the exposure of functionalized graphene material to the readily oxidized condition [21]. High-resolution N1s spectrum of FG-CYS (Figure 3c), on the other hand, exhibited several peak components including peaks at 400.0 eV, 401.1 eV, 401.7 eV and 402.5 eV, which can be allotted to C–NH_2_, NH_3_^+^, N–H and C–N^+^, respectively [8,18,22]. The presence of the nitrogen species from the N1s peak deconvolution analysis was in good correlation with the chemical structure of the thiol precursor, L-cysteine ethyl ester, that corroborated successful attachment of the thiol precursor on the surface of graphene [13,16,23]. These results were well-supported by the high-resolution C1s spectra of FG-CYS (Figure S2), with additional peak components of C–S, C–N, C=O and O–C=O identified at binding energies of 285.4 eV, 286.1, 287.7 eV and 288.6 eV, respectively, besides the typical C=C (284.5 eV) and C–C (284.8 eV) peaks.

Detailed Raman analysis was performed to verify the modification created on the surface of pristine graphene and functionalized graphene, as illustrated in Figure 3d–e. Both of the samples showed the main features of G band at around 1580 cm^−1^, D band at around 1350 cm^−1^, and 2D band at 2700 cm^−1^ [24,25]. The presence of graphene can be confirmed with a sharp G peak (~1580 cm^−1^) and a symmetrical 2D peak (~2700 cm^−1^) as clearly observed on the Raman spectra of Gr and FG-CYS samples. As depicted in Figure 3d, an indicative D’ peak (indicated by the black arrow) for defective graphene positioned at ~1620 cm^−1^ was detected on the Raman spectrum of FG-CYS, suggesting a certain degree of defect found on the surface of FG-CYS. This finding was in consistent with the average value of I_D_/I_G_ ratio (a measurand of the level of disorder of graphene) of FG-CYS (0.7), which was higher than Gr (0.3), indicating more defects were experienced on the surface of FG-CYS than Gr due to thiol-ene click modification using thiol precursor, as shown in Figure 3e [26]. The level of disorder after the modification using thiol precursor was also evidenced by the broadening of the 2D and G peaks, as attested by the significant increment of the full-width at half-maximum of the 2D peak and G peak, FWHM [2D] and FWHM [G] [27,28]. As shown in Figure 3e, the dramatic increase from 20.2 to 35.5 (FWHM [G]) and 64.8 to 90.1 (FWHM [2D]) for Gr to FG-CYS, respectively, clearly indicated that substantial defects were introduced to the surface of graphene that could have resulted from the thiol-ene click reaction.

The obtained FTIR characterization results presented in Figure 3f were further used to elucidate the functional groups attached on the surface of the functionalized graphene. A nearly flat FTIR curve with no obvious peak was detected for Gr sample due to the lack of chemical functional groups on its surface. Essentially, a band representing the C–S vibration was visible at around 600–800 cm^−1^ on the FTIR spectrum of the functionalized material, but not identified on the FTIR spectrum of pristine graphene [29]. This finding was consistent with the result from XPS analysis with a substantial amount of S detected and C–S–C species found in the functionalized sample, after the modification process using thiol precursor. The absence of a peak at around 2500 cm^−1^ (stretching of S–H group) in the functionalized graphene suggested that all the –SH groups in the thiol precursor had reacted with the sp^2^ carbon in pristine graphene through thiol-ene click reaction [29]. Additionally, the successful modification of the pristine graphene was also evidenced by the appearance of the peak at approximately 1389 cm^−1^ for the stretching of C=O in the ester group on FG-CYS. Peaks at about 1640 cm^−1^ (N–H bending), 1090 cm^−1^ (C–N stretching) and 1054 cm^−1^ (C–NH2 vibration) were also found on the FTIR spectrum of FG-CYS, implying successful grafting of the functional groups on the pristine graphene. Two common peaks at 2913 and 2846 cm^−1^, which can be attributed to the asymmetric and symmetric vibrations of C–H of –CH_2_ group, respectively, were also observed in the functionalized graphene [29,30]. Based on the decoration of the functional groups on the functionalized graphene, as elucidated from the FTIR analysis, which was well-correlated with the XPS analysis discussed in the previous section, we can infer that the successful click of thiol precursor to the pristine graphene has occurred through the thiol-ene pathway.

Furthermore, TGA was also conducted on pristine and functionalized graphene to qualitatively and quantitatively determine the presence of attached functional groups on the pristine graphene after the thermal thiol-ene click reaction. As presented in Figure 3g, the TGA curves (solid blue and red lines) clearly showed a distinguishable mass loss pattern experienced by Gr and FG-CYS. Pristine graphene showed higher thermal stability with only 7.28% total mass loss, relative to its functionalized graphene that experienced an overall 16.41% mass loss when heated under inert atmosphere to 1000 °C. This result was consistent with the high thermal stability of the typical pristine graphene found in the literature [31,32,33]. From the DTG curve of the functionalized graphene, two major mass loss steps were identified at about 250 °C and 400 °C, apart from a small mass loss (0.52%) that was accountable for the elimination of water below 100 °C. The first mass loss step could be associated with the detachment of the oxygen functional groups, including ester from the graphene surface [8]. Meanwhile, the second mass loss at around 400 °C can be related to the cleavage of the remaining covalently bound groups, such as oxygen, sulfur, and amino moieties from the functionalized graphene surface. From the TGA curves of the control and functionalized graphene, the grafting density of the modified sample can be estimated by calculating the value of functional group coverage (average carbon atom number containing functional group) based on the mass loss difference of FG-CYS from Gr, as summarized in Table S1 [15,16]. Hence, it can be estimated that 1 cysteine molecule per 113 carbon atoms was grafted on the graphene sheets in this thiol-ene click modification, as quantitatively determined from the TGA.

Despite the key challenges of inertness of sp^2^ carbon and the lack of reactive functional groups on the surface of pristine graphene, we successfully demonstrated an effective functionalization of pristine graphene through a green and scalable thiol-ene click modification to endow a highly dispersible graphene. A summary of our key findings with evidence supported by comprehensive techniques, as tabulated in Table 2, confirmed the proposed methodology as a powerful tool for the preparation of dispersible and functional graphene materials with promising applications across different arenas, including biomedical, coatings, energy storage and environment. In the next section, we compare and discuss the degree of functionalization of FG-CYS with similar functionalized graphene prepared in the literature.

## 4. Discussion

To date, functionalization of pristine graphene by covalent bonding has rarely been reported in relation to its derivative, GO, due to the lack of reactive functional groups on the pristine graphene surface [5]. It is undeniable that functionalized graphene has several limitations, including poor dispersion in water, low functional group coverage, and high toxicity of solvent used during the functionalization process. Click chemistry introduced by Sharpless et al. can be regarded as a promising solution to overcome the chemical inertness of C=C bonds in the graphene framework to afford a highly dispersible graphene, despite the fact that it is often accompanied by low reaction efficiency [6,15].

Numerous attempts have been made in the last decade to introduce organic functional groups onto the surface of pristine graphene through thiol-ene click chemistry. The degree of functionalization can be quantitatively gauged using both bulk (TGA) and surface (XPS) characterization techniques, as summarized in Table 3. To perform an impartial comparison, the value of the functional group coverage estimated from the mass loss through TGA method, and the chemical composition of specific element such as N or S in the thiol precursor determined through XPS analysis, were compared to determine the level of thiol-ene functionalization.

Based on the bulk TGA characterization technique, L-cysteine was found to be more effectively grafted on FG-CYS (present study) than G-LCHa, as reflected by its lower functional group coverage of 113, compared to 137 carbon atoms per unit of L-cysteine molecule, as tabulated in Table 3. Further surface elemental quantification analysis (XPS) showed that only 0.89 at % N and 0.7 at % S were detected on G-LCHa that was modified using microwave-assisted thiol-ene click reaction; while 6.26 at % N and 4.79 at % S were identified on the FG-CYS produced through the thermal thiol-ene click approach in our present study [15]. 

Another example of direct functionalization on pristine graphene was manifested in G-CHI and GR-Cys, with 1 cysteamine molecule per 113 and 96 carbon atoms, respectively, based on the value of functional group coverage. Meanwhile, 1.13 at % N and 1.66 at % S were found on G-CHI, while 4.78 at % N and 4.52 at % S were detected on GR-Cys after the thiol-ene click modification [15,16]. In comparison, FG-CYS (our present study) appeared to outperform G-CHI but slightly underperformed compared to GR-Cys. Note that several aspects including safety, scalability and economy feasibility should be considered when dealing with the use of a functionalization method. The majority of the thiol-ene functionalized graphene was produced using toxic solvents such as NMP and THF, which are not environmentally friendly and could potentially trigger chemical risk in the working environment. With respect to the environmental hazard, energy consumption, cost and scalability of producing dispersible graphene, the green thiol-ene click functionalization approach developed in the present work still surpassed the thiol-ene functionalized graphene reported in the literature based on the functionalization condition applied and the effective functionalization degree achieved by FG-CYS.

Despite the growing body of work recently reported to functionalize graphene directly using thiol-ene click strategy, there are still numerous open research opportunities to address the graphene dispersion issue, to endow a better processability of graphene materials (new functionalized graphene) with desired properties. Beyond the conventional chemical modification of graphene such as the thermal heating process, other methods including microwave irradiation, plasma etching, electrochemical modification or a combination of these methods could be explored as promising alternative routes to resolve the low reactivity of graphene. Based on our current work, functionalization parameters such as the ratio of graphene to thiol precursor, reaction time and temperature, could be tuned to accommodate a more effective grafting of functional groups on the graphene surface. The future direction of graphene functionalization may be structured towards room temperature modification of graphene, which can be feasibly achieved via the photoinitiated thiol-ene click approach. In addition, more research efforts should be dedicated to the optimization of functionalization methods to achieve not only the better dispersion of graphene, but also the scalability of processing of new graphene materials to be more economical.

## 5. Conclusions

In summary, the synthesis of functionalized pristine graphene by a green thermal thiol-ene click approach using L-cysteine ethyl ester was successfully demonstrated. The use of ethanol in replacing highly toxic organic solvents in this click reaction provides a generic, green, and scalable approach to graft graphene with desired functional groups, which can be achieved by selecting different types of thiol precursors bound with functional groups including hydroxyl, ester and amine groups. Successful modification of pristine graphene with the thiol precursor was confirmed by Raman, FTIR, TGA and XPS analyses. This green modification process provides a significant contribution towards the scalable production of functionalized graphene due to a simple and scalable chemical route which is beneficial for many applications, such as biomedical, sensing, graphene polymer composites, inks, supercapacitors, etc.

## Figures and Tables

**Table 1:** Normalized atomic percentage of elements determined from the XPS survey scan of Gr and FG-CYS.

Sample/Element	Atomic Composition (±0.3%)			
	C	O	S	N
Gr	91.60	8.40	N.A.	N.A.
FG-CYS	71.65	17.30	4.79	6.26

**Table 2:** Summary of thiol-ene click functionalization of FG-CYS as evidenced by selected key techniques.

Sample	Water Dispersion	XPS (% S)	Raman (I_D_/I_G_ Ratio)	FTIR (Attached Functional Groups)	TGA (Functional Group Coverage *)
Gr	Less than 2 h	N.A.	0.3	N.A.	N.A.
FG-CYS	More than a week	4.79	0.7	N–H, C–NH_2_, C=O, C–S	113 *

* Detailed calculation can be found in the Supplementary Material.

**Table 3:** Comparison of grafting density based on surface (XPS) and bulk (TGA) quantification techniques.

Sample	Modification Method	Solvent	Thiol Precursor	TGA (Mass Loss %); Functional Group Coverage	XPS at % (N; S)	References
G-LCHa	Microwave-assisted thiol-ene click (70 °C)	Tetrahydrofuran (THF)	L-cysteine	8.58; 137	0.89; 0.7	[15]
G-CHI	Microwave-assisted thiol-ene click (70 °C)	Tetrahydrofuran (THF)	cysteamine	7.60; 113	1.13; 1.66	[15]
GR-Cys	Thermal thiol-ene click (70 °C, 7 h)	N-methyl-2-pyrrolidone (NMP)	cysteamine	15.80; 96	4.78; 4.52	[16]
FG-CYS	Thermal thiol-ene click (65 °C, 24 h)	Ethanol–water	L-cysteine ethyl ester	8.61; 113 *	6.26; 4.79	This work

* Detailed calculations can be found in the Supplementary Material.

## Data Availability

The data presented in this study are available on reasonable request from the corresponding author.

## Supplementary Materials

The following are available online at https://www.mdpi.com/article/10.3390/ma14112830/s1, Figure S1: Histogram on the number of layers of Gr, showing an average six layers of graphene sheets with standard deviation determined from 20 measurements by HRTEM analysis, Figure S2: XPS high-resolution C1s plots of Gr and FG-CYS, Table S1: Results of TGA and the value of functional group coverage of FG-CYS estimated from TGA.
