# Supplementary material for: Highly Water Dispersible Functionalized Graphene by Thermal Thiol-Ene Click Chemistry
Source: Materials (Basel). 2021 May 25;14(11):2830. doi: 10.3390/ma14112830 (PMC8198566; doi:10.3390/ma14112830)
Supplement: Supplementary file 1 [file materials-14-02830-s001.zip › materials-1212894-supplementary.pdf]

materials

Supplementary Materials

Highly Water Dispersible Functionalized Graphene by Thermal Thiol-Ene Click Chemistry

Farzaneh Farivar [1,2], Pei Lay Yap[1,2], Tran Thanh Tung[1,2] and Dusan Losic [1,2,*]

[1] School of Chemical Engineering and Advanced Materials, The University of Adelaide, Adelaide, SA 5005, Australia; farzaneh.farivar@adelaide.edu.au (F.F); peilay.yap@adelaide.edu.au (Y.P.L.); tran.tung@adelaide.edu.au (T.T.T.)
[2] ARC Hub for Graphene Enabled Industry Transformation, The University of Adelaide, Adelaide, SA 5005, Australia
* Correspondence: dusan.losic@adelaide.edu.au

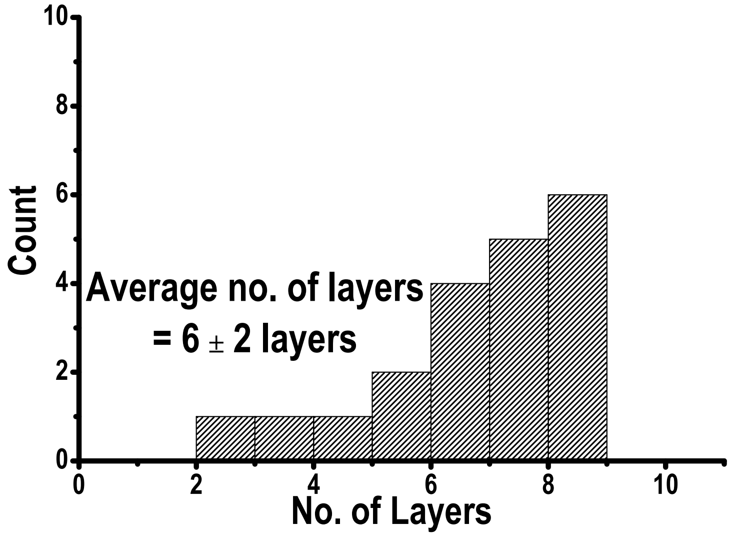

Figure S1. Histogram on the number of layers of Gr, showing an average six layers of graphene sheets with standard deviation determined from 20 measurements by HRTEM analysis.

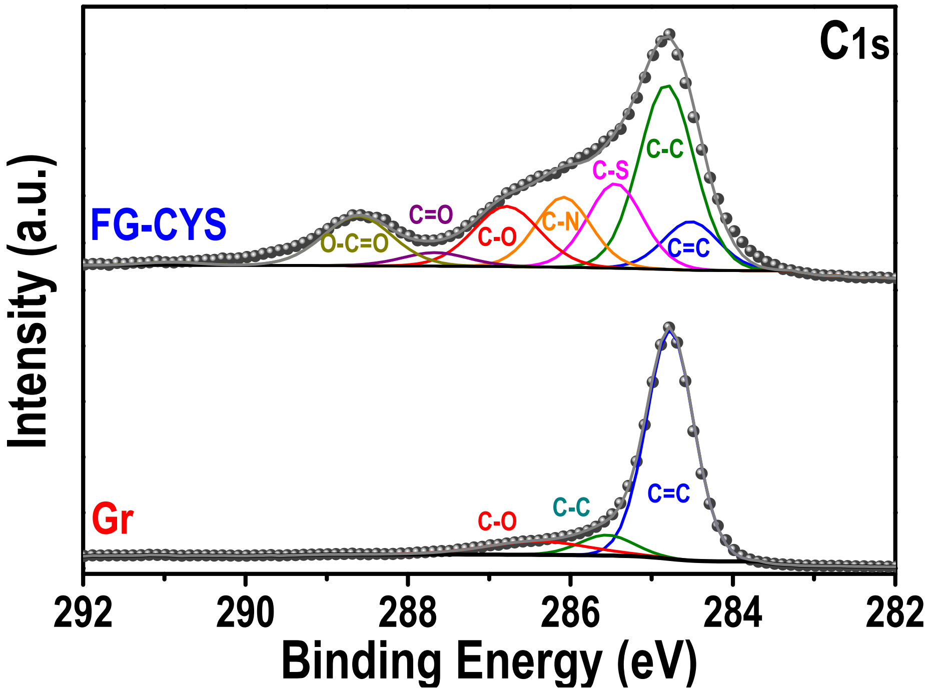

Figure S2. XPS high resolution C1s plots of Gr and FG-CYS.

Table 1. Results of TGA analysis and the determination of functional group coverage of FG-CYS estimated from TGA analysis.

Sample	Gr	FG-CYS
**Total mass loss (%)**	7.28	16.41
**Residual mass (%)**	92.72	83.59
**Mass loss < 100 °C (%)**	N.A.	0.52
**Mass loss w.r.t. CYS (%)**	N.A.	8.61
**Initial mass (mg)**	4.7423	4.304
**Molar mass (g/mol)**	C: 12	L-cysteine ethyl ester: 149.21
**No. of atoms/molecules**	2.00×10^{20}	1.77×10^{18}
**Functional group coverage***	N.A.	113

* **Functional group coverage** can be estimated as follows [1,2]:

1. The number of CYS molecule was calculated using its molar mass and mass loss of CYS determined from TGA analysis.
2. The number of carbon atoms in graphene can be determined based on the remaining TGA mass of graphene and its molar mass.
3. Functional group coverage can be estimated by dividing the number of carbon atoms in graphene by the number of CYS molecule.

References

1. Peng, Z.; Li, H.; Li, Q.; Hu, Y. Microwave-Assisted thiol-ene click chemistry of carbon nanoforms. *Colloids Surf., A* **2017**, *533*, 48–54, doi:10.1016/j.colsurfa.2017.08.018.
2. Li, Y.; Bao, L.; Zhou, Q.; Ou, E.; Xu, W. Functionalized Graphene Obtained via Thiol-Ene Click Reactions as an Efficient Electrochemical Sensor. *ChemistrySelect* **2017**, *2*, 9284–9290, doi:10.1002/slct.201700659.
